# Balancing Selection of a Frame-Shift Mutation in the *MRC2* Gene Accounts for the Outbreak of the Crooked Tail Syndrome in Belgian Blue Cattle

**DOI:** 10.1371/journal.pgen.1000666

**Published:** 2009-09-25

**Authors:** Corinne Fasquelle, Arnaud Sartelet, Wanbo Li, Marc Dive, Nico Tamma, Charles Michaux, Tom Druet, Ivo J. Huijbers, Clare M. Isacke, Wouter Coppieters, Michel Georges, Carole Charlier

**Affiliations:** 1 Unit of Animal Genomics, GIGA-R, Department of Animal Sciences, Faculty of Veterinary Medicine, University of Liège, Liège, Belgium; 2 Unit of Bioinformatics, Department of Animal Sciences, Faculty of Veterinary Medicine, University of Liège, Liège, Belgium; 3 Breakthrough Breast Cancer Research Centre, The Institute of Cancer Research, London, United Kingdom; Stanford University School of Medicine, United States of America

## Abstract

We herein describe the positional identification of a 2-bp deletion in the open reading frame of the *MRC2* receptor causing the recessive Crooked Tail Syndrome in cattle. The resulting frame-shift reveals a premature stop codon that causes nonsense-mediated decay of the mutant messenger RNA, and the virtual absence of functional Endo180 protein in affected animals. Cases exhibit skeletal anomalies thought to result from impaired extracellular matrix remodeling during ossification, and as of yet unexplained muscular symptoms. We demonstrate that carrier status is very significantly associated with desired characteristics in the general population, including enhanced muscular development, and that the resulting heterozygote advantage caused a selective sweep which explains the unexpectedly high frequency (25%) of carriers in the Belgian Blue Cattle Breed.

## Author Summary

Livestock are being subject to intense artificial selection aimed at ever-increasing, sometimes extreme, production phenotypes. This is well-illustrated by the exceptional muscular hypertrophy characterizing the “double-muscled” Belgian Blue Cattle Breed (BBCB). We herein identify a loss-of-function mutation of the bovine *MRC2* gene that increases muscle mass in heterozygotes, yet causes skeletal and muscular malformations known as Crooked Tail Syndrome (CTS) in homozygotes. As a result of the “heterozygote advantage”, the *MRC2 c.2904_2905delAG* mutation has swept through the BBCB population, resulting in as many as 25% carrier animals and causing a sudden burst of CTS cases. These findings highlight one of the risks associated with pushing domestic animals to their physiological limits by intense artificial selection.

## Introduction

The Belgian Blue Cattle breed (BBCB) is notorious for its exceptional muscular development known as “double-muscling”. This extreme phenotype is due in part to an 11-bp loss-of-function deletion in the myostatin gene that has been fixed in the breed (e.g. [1]), as well as to ongoing selection on as of yet unidentified polygenes influencing muscularity. As in other breeds, intense selection has substantially reduced the effective population size. Extensive reliance on artificial insemination (AI), in particular, by allowing popular sires to have thousands of descendants, narrows the genetic basis. The concomitant increase in the rate of inbreeding causes recurrent outbreaks of recessive defects. Inherited defects that have lately afflicted the BBCB include the recently described Congenital Muscular Dystonias (CMD) I and II [2].

As a result of this peculiar demography of domestic animal populations, inherited defects generally involve unique “founder” mutations. Allelic homogeneity greatly facilitates positional identification using identity-by-descent (IBD) mapping, as recently demonstrated using the first generation high density SNP arrays for the bovine [2]. The genes underlying CMD I & II were readily mapped, and the causative mutations in the *ATP2A1* and *SLC6A5* genes identified. The widespread use of the resulting diagnostic tests allowed immediate and effective control of the corresponding pathologies.

We herein report the positional identification of the mutation causing a novel, recently appeared defect referred to as Crooked Tail Syndrome (CTS). The incidence of CTS has risen very suddenly in the BBCB, and 25% of animals now appear to be CTS carriers. We herein provide strong evidence for exacerbated muscular development of carriers of the CTS mutation, conferring “heterozygote advantage” underlying the selective sweep that raised the causative mutation to alarming proportions.

## Results

### Crooked Tail Syndrome (CTS) exhibits variable expressivity

**Figure 1:**
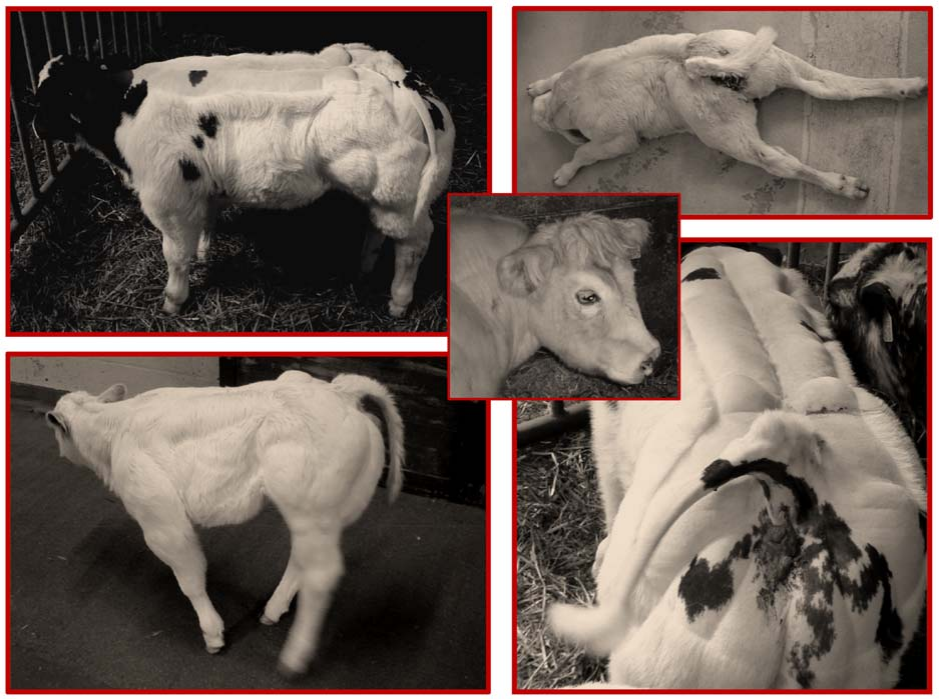
Clinical spectrum exhibited by CTS cases. Crooked tail, growth retardation, stocky head, extreme muscular hypertrophy, spastic paresis of the hind limbs, straight hock, scoliosis.

We recently established a heredo-surveillance platform operating in close collaboration with field veterinarians to rapidly identify emerging genetic defects. As part of these activities, 105 CTS cases were reported to the platform between November 2006 and November 2007. In addition to the striking deviation of the tail (equally likely to be dextro- or levo-rotatory), detailed clinical examination revealed three symptoms shared by all cases: (i) general growth retardation manifesting itself at approximately one month of age, (ii) abnormal skull shape manifested as a shortened broad head, and (iii) extreme muscular hypertrophy including a conspicuous outgrowth of the gluteus medius anchor. Additional symptoms were observed in a substantial proportion but not all cases: (i) spastic paresis of the hind limbs affecting either the quadriceps only (22%), or quadriceps and gastrocnemius (14%), often associated with straight hocks, (ii) short, straight and extended fore limbs (33%), and (iii) pronounced scoliosis with asymmetric development of the muscles of the back (20%). Figure 1 illustrates the corresponding symptomatology. We performed complete necropsy of a few selected cases but detected no additional obvious abnormalities. Moreover, radiological examination of crooked tails and scoliotic spines failed to reveal structural defects of the vertebrae (data not shown).

Although the defect is not lethal by itself, the most severe cases (∼25%) were euthanized on welfare grounds. The surviving ∼75% nevertheless caused important economic losses to their owners as a result of growth retardation and carcass depreciation.

### CTS is caused by a fully penetrant, two base pair deletion in the open reading frame (ORF) of the *MRC2* gene

**Figure 2:**
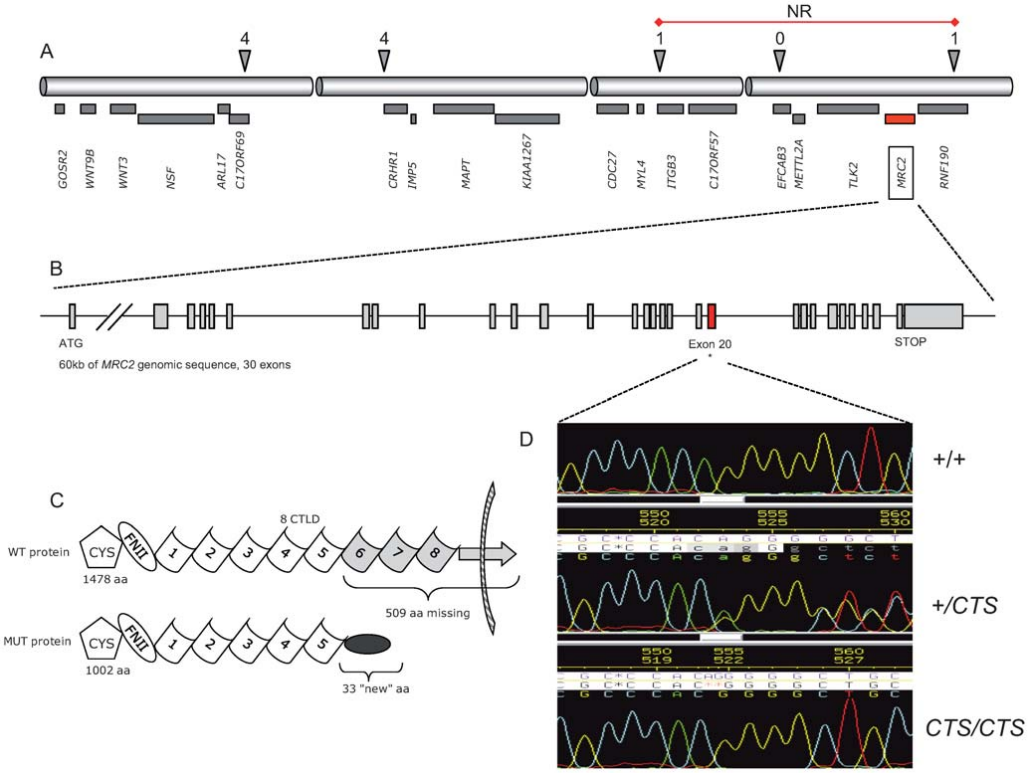
Positional identification of the *c.2904_2905delAG MRC2* mutation causing CTS, and its effect of the Endo180 protein. (A) Gene content of the 2.4 Mb interval in which the CTS mutation was located using identity-by-descent mapping. The triangles correspond to five SNPs used to refine the CTS locus position, with corresponding number of recombinant individuals out of 105 CTS cases. The resulting non-recombinant (NR) interval is marked by the red horizontal line. (B) Structure of the *MRC2* gene within that interval. (C) Domain composition of the wild-type (WT) and mutant (MUT) Endo180 protein. (D) Sequences traced obtained from genomic DNA of a homozygous wild-type, carrier and homozygous CTS animal showing the deletion of the ApG dinucleotide in the mutant.

We previously mapped the CTS locus to bovine chromosome 19, in a 2.4 Mb interval shared homozygous-by-descent by the eight analyzed CTS cases [2]. To refine the map location of the CTS locus we genotyped the 105 reported CTS cases for five SNPs covering the 2.4 Mb interval (Figure 2A). The SNPs were selected on the basis of the low population frequency of the disease-associated allele. Genotyping was achieved by first sequencing 35 pools of three animals, followed by individual sequencing of the pools revealing the presence of the major allele and therefore of one or more recombinants. This approach allowed us to confine the critical region to the 812 Kb rs29010018 - AAFC03034831 interval. It comprises seven annotated genes which were ranked on the basis of their perceived relevance with regard to the CTS condition. Coding exons were sequenced in an affected and a matched healthy control individual.

During this process, we identified a 2-bp deletion in the ORF of the mannose receptor C type 2 (*MRC2*) gene. The *MRC2* gene encodes the 180 kDa Endocytic Transmembrane Glycoprotein (Endo180), one of the four members of the mannose receptor family [3],[4]. Endo180 is a recycling endocytic receptor that is predominantly expressed in mesenchymal cells such as stromal fibroblasts and in the chondrocytes and osteoblasts/osteocytes in the developing bones, and is proposed to play a role in regulating extracellular matrix degradation and remodelling. It has C-type lectin activity, binds collagen and interacts with urokinase-type plasminogen activator receptor (uPAR) in a trimolecular cell surface complex with pro-urokinase plasminogen activator (pro-uPA). The 180 kD Endo180 protein comprises an aminoterminal cysteine-rich domain of unknown function, a fibronectin type II domain which mediates collagen binding, eight C-type lectin-like domains (CTLDs) of which the second mediates Ca^2+^-dependent lectin activity, a stop-transfer signal anchoring this single-pass transmembrane protein in the membrane, and a carboxyterminal cytoplasmic domain allowing association with adaptor proteins in the clathrin coat. The mutation identified in CTS cases is located in exon 20 and deletes nucleotides 2904 and 2905 of the *MRC2* cDNA (*c.2904_2905delAG*). It is predicted to append a frame-shifted 30-residue peptide to a truncated Endo180 receptor missing the CTLD6-8 domains, the stop-transfer signal and the cytoplasmic domain (Figure 2B,C,D). As a result, the mutated protein should be unable to localize to the plasma membrane and mediate receptor-mediated endocytosis.

We developed a 5′ exonuclease assay for the mutation and genotyped the 105 reported CTS cases. All proved to be homozygous for the *c.2904_2905delAG* mutation. We then genotyped 1,899 healthy Belgian Blue animals. Unexpectedly, 24.7% of animals appeared to be carriers, without a single homozygous mutant (p<10^−12^ assuming Hardy-Weinberg equilibrium). Taken together, these results allowed us to incriminate the *c.2904_2905delAG* mutation as being causal and fully penetrant.

### Mutant *MRC2* mRNAs are targeted by the nonsense-mediated decay (NMD) RNA surveillance pathway

**Figure 3:**
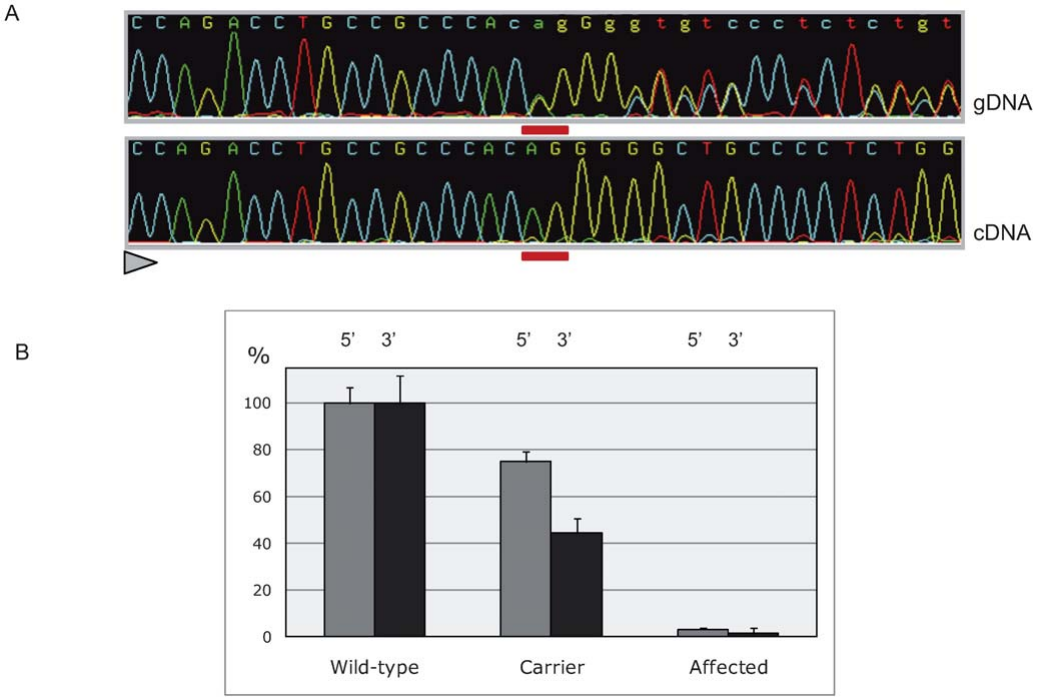
Nonsense-mediated RNA decay of *c.2904_2905delAG* mutant *MRC2* transcripts. (A) Direct sequencing of *MRC2* amplicons spanning the CTS mutation obtained from genomic DNA and pulmonary cDNA of a heterozygous animal, showing the virtually exclusive detection of wild-type allele amongst transcripts (the position of the deleted nucleotides is underlined in red, the sequencing direction is represented by a triangle). (B) Comparing *MRC2* mRNA levels in the lung of +/+, +/CTS and CTS/CTS animals. Data are shown for two amplicons at the 5′ and 3′ ends of the mRNA, respectively. Error bars correspond to standard errors over three replicates per sample.

*C.2904_2905delAG* causes a frame-shift resulting in a premature stop codon in the 21^st^ of the 30-exon *MRC2* gene. Mutant mRNAs are therefore predicted to undergo NMD [5]. To test this, we first compared the levels of wild-type and mutant *MRC2* mRNA in lung and skeletal muscle of a carrier animal by direct sequencing of RT-PCR products encompassing the deletion. As can be seen from Figure 3A, mutant mRNA was barely detectable. We then compared the levels of *MRC2* mRNA in lung tissue of animals of the three genotypes using quantitative RT-PCR performed with primer sets targeting the 5′ and 3′ end of the mRNA respectively. Highly significant reductions in *MRC2* mRNA levels were observed in carriers relative to homozygous wild-type individuals (75%±4% and 45%±6% of control values for the 5′ and 3′ systems respectively), while *MRC2* mRNA levels in cases were less than 5% of homozygous wild-types (Figure 3B). Both the allelic imbalance and qRT-PCR experiments thus supported degradation of the mutant transcripts by NMD.

### Amounts of full-length Endo180 protein are halved in tissues of carrier animals

**Figure 4:**
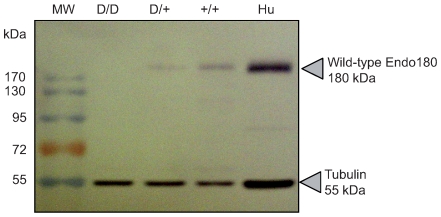
Effect of the CTS mutation on the levels of full-length Endo180. Western blot results from lung of animals of the three genotypes. Hu: human control sample. MW: molecular weight marker. The 55 Kd band corresponds to non-specific binding of the CAT2 antibody to tubulin, used as control for the amount of protein loaded.

From the ten anti-human Endo180 antibodies tested by Western blotting, only one polyclonal rabbit antibody (CAT2) detected the bovine Endo180 protein with sufficient specificity. The CAT2 antibody is directed against the last 19 amino acids of Endo180 of which the last 18 are perfectly conserved between human and cow [6]. CAT2 was thus predicted to allow recognition of the wild-type but not mutant Endo180. As expected, no wild-type Endo180 was detected in lung tissue of CTS affected animals. In carrier animals, the levels of Endo180 protein were approximately half those observed in homozygous wild-types (Figure 4). Assuming that Endo180 is dosage sensitive, such reduction in the supposedly functional species might affect phenotype.

### CTS carrier status increases muscle mass

The unusually high frequency of the CTS mutation in BBCB suggested that it might confer heterozygote advantage in this highly selected population. To test this hypothesis we estimated the effect of carrier genotype on 22 type traits evaluating muscularity, skeletal conformation, size and leg soundness, which are systematically recorded as part of the selection programs implemented in the BBCB. The analysis was conducted on 519 pedigreed bulls, including 148 carrier and 371 homozygous wild-type animals, using a mixed model including fixed effects of *MRC2* genotype, year at scoring, body condition and age at scoring, as well as a random individual animal effect. Variance components and effects were estimated by restricted maximum likelihood (REML) analysis. Highly significant effects were obtained for the four categories of recorded traits (Table 1). CTS carrier animals were smaller, stockier and more heavily muscled. They had a thinner skeleton and more rounded ribs, which are characteristics of beef cattle. *MRC2* genotype accounted for 3.6%, 3.6% and 2.6% for the genetic variance of height, muscularity and general appearance, respectively. These results strongly suggest that CTS carrier frequency is increased by selection programs applied in BBCB.

**Table 1:** Effect of CTS carrier status on type traits in BBCB.

	Trait or syntetic note	Contrast	Std. error	p value	Carrier characteristics
**SIZE**	Withers height	2.33	0.320	***	Smaller
	Length	0.36	0.181	N.S.	
	Chest width	−0.68	0.319	*	Larger
	Pelvis width	−0.32	0.188	N.S.	
	Pelvis length	0.25	0.163	N.S.	
**MUSC.**	Shoulder muscling	−0.58	0.283	*	Increased muscularity
	Top muscling	−1.71	0.409	***	Increased muscularity
	Buttock side	−0.35	0.20	N.S.	
	Buttock rear	−0.50	0.251	*	Increased muscularity
	Synthetic note for muscularity	−0.70	0.246	**	Increased muscularity
	General appearance	−1.73	0.124	***	Better
**SKELETAL CONFORM.**	Skeleton	−1.18	0.386	**	Thinner
	Rib shape	−1.67	0.456	***	Ronder
	Fore legs stance	0.54	0.157	***	More toed-in
	Rear legs stance	−0.43	0.200	*	More toed-out
**OTHER**	Skin	−0.34	0.442	N.S.	
	Tail set	−0.24	0.529	N.S.	
	Shoulder bone	0.10	0.108	N.S.	
	Rump	1.25	0.425	**	More horizontal
	Top line	−0.36	0.137	**	More convex
	Hocks stance	0.84	0.367	*	Straighter

* p<5%, ** p<1%, *** p<1%.

### The *C.2904_2905delAG* mutation is undergoing a selective sweep

To more directly demonstrate the occurrence of a selective sweep, we performed the following analysis. Examination of the available genealogies of the 105 affected individuals indicated that all of them trace back to Précieux, a popular AI sire, via both sire and dam. This suggested that Précieux, born in 1980, was CTS carrier and that its extensive utilization in the mid eighties spread the CTS mutation in BBCB. Genotyping Précieux and three of his sons for the *C.2904_2905delAG* mutation and the 60K Illumina chip, indeed demonstrated that he carried the CTS mutation embedded in the SNP haplotype shared homozygous-by-descent by the examined cases [2]. Thus, the vast majority of *C.2904_2905delAG* mutations encountered in present-day BBCB animals, trace back to Précieux.

We obtained DNA samples from all BBCB sires (174) born between 2003 and 2005, whose semen had been commercialized by one of the ten major Belgian AI studs. Such AI sires are heavily selected for extreme muscularity. Examination of the pedigrees indicated that 160 of the 174 [2003–2005] AI sires were descendents of Précieux. The number of generations separating these sires from Précieux averaged 5.9 (range: 3 to 8). Genotyping the *C.2904_2905delAG* mutation in this cohort identified 45 CTS carriers, all of them amongst the 160 descendents of Précieux.

Assuming that the CTS mutation indeed underwent a selective sweep, 45 carriers out of the 160 Précieux descendants would be significantly higher than expected by chance alone. To verify this assumption we simulated the segregation of a mutation in the true genealogy of the 160 descendants of Précieux and counted the resulting number of carrier bulls. In these simulations, Précieux was systematically assumed to be carrier, while the frequency of the mutation in animals unrelated to Précieux varied from 0 to 0.05. In the absence of selection (i.e. if a carrier animal is equally likely to transmit either the mutation or the wild-type allele to anyone of its descendents), the probability to obtain 45/160 carriers was 0.0014, 0.0023 and 0.0130 for mutation frequencies (outside the Précieux lineage) of 0.00, 0.01 and 0.05, respectively (Table S1). Thus we can confidently assert that the *C.2904_2905delAG* mutation indeed underwent a selective sweep in the BBCB.

**Figure 5:**
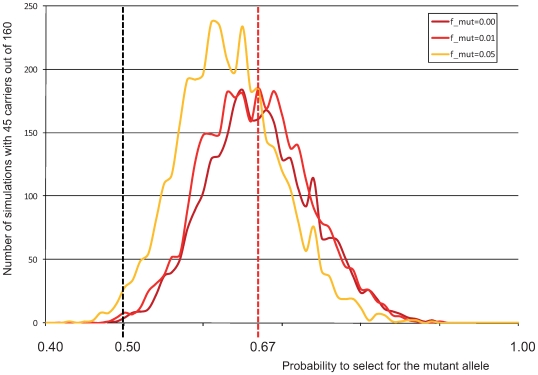
Distribution of the number of simulations (out of 10,000) yielding 45 carriers out of 160 Précieux descendants (Y-axis), as a function of the rate of transmission of the mutation from heterozygous carriers (X-axis). Three curves are given corresponding to frequencies of the mutation outside of the Précieux lineage of 0, 1, and 5%. The dotted red vertical line corresponds to a transmission rate of 67%, maximizing the number of simulations yielding 45 carriers for a mutation frequency (outside of the Précieux lineage) of 1%, considered to be an upper bound in BBCB.

To have some quantitative assessment of the intensity of the selective sweep, we repeated the “gene dropping” simulations while varying the degree of segregation distortion in favour of the mutant allele. Figure 5 shows the proportion of simulations yielding 45/160 carrier bulls as a function of the transmission probability of the CTS mutation from carrier parents to offspring. It can be seen that the outcome of 45/160 carrier bulls is most likely for a transmission rate between 0.62∶0.38 (mutation frequency outside Précieux lineage of 0.05) and 0.67∶0.33 (mutation frequency outside Précieux lineage ≤0.01). The fact that all 105 CST cases traced back to Précieux both on the dam and sire side, indicates that the mutation frequency outside of the Precieux lineage is closer to 1% than to 5%. Thus, a carrier animal is approximately two times more likely to be selected than a non-carrier sib.

## Discussion

We herein describe a frame-shift mutation in the *MRC2* gene causing the CTS syndrome in cattle. Clinical manifestations of CTS are dominated by skeletal and muscular anomalies. Skeletal symptoms including growth retardation, abnormally shaped legs and skulls, are perfectly compatible with the known involvement of *MRC2* in regulating extracellular matrix degradation and remodeling and its strong expression in developing bone [7]. The muscular symptoms, including muscular hypertrophy, tail deviation and spastic paresis are more difficult to rationalize, although a role for the related mannose receptor in myoblast motility and muscle growth has been recently reported [8]. We cannot formally exclude the possibility that the muscular manifestations result from distinct sequence variants in linkage disequilibrium with the CTS mutation, although we favor the more parsimonious hypothesis of a single causative mutation.

It is noteworthy that mice homozygous for a targeted deletion of *MRC2* exons 2 to 6 have been generated in two independent laboratories [9],[10]. Both laboratories reported that the mice were viable and fertile, although more recently a minor deficiency in long bone growth, bone mineral density and calvarial bone formation has been demonstrated [7]. Cells derived from these animals show a clear defect in collagen uptake and degradation. One reason for the more pronounced clinical manifestations in cattle than in mice may lie in the distinct nature of the murine and CTS *MRC2* mutations. Cells isolated from the genetically modified mice express a mRNA species in which exon 1 (containing the signal sequence) is spliced in frame onto exon 7 (containing CTLD2), and in embryonic fibroblasts a truncated Endo180 protein missing the cysteine-rich, FNII and CTLD1 domains can be expressed [9]. However, little or no truncated protein is found in postnatal tissues from these knockout mice [10],[11]. Alternatively it may be that there are distinct degrees of redundancy between members of the mannose receptor family in different species. Also the more striking phenotype in cattle may be due the different genetic background and particularly the fact that the studied animals were all homozygous for a *MSTN* loss-of-function mutation [1]. This hypothesis could be tested by mating the available *MRC2* and *MSTN* knock-out mice. Whatever the reason, at this point bovine CTS may be the more informative model to decipher *MRC2* function, and to assist in the identification of as of yet unidentified human pathological conditions resulting from *MRC2* loss-of-function.

We provide very strong evidence of phenotypic manifestations of the CTS mutation in carriers. This is more than likely reflecting dosage sensitivity for Endo180, as NMD causes the mutant protein to be present at near undetectable levels, thus very unlikely to affect cellular function *per se*. Enhanced muscularity of CTS carriers has supposedly contributed greatly to the rapid increase of the CTS mutation in the BBCB. Indeed, we demonstrate that carrier animals have approximately two times more chance to be selected as elite sires than their non-carrier sibs. Note that this is the level of segregation distortion expected for a gene that accounts for ∼5% of the genetic variance for a trait with heritability of ∼25% and assuming a selection intensity of ∼2% (Table S2).

Such selective sweep is reminiscent of the spread of other inherited defects in domestic animals as a result of advantageous traits exhibited by carriers. These include loss-of-function mutations of the porcine ryanodine receptor and equine skeletal muscle sodium channel alpha subunit gene causing, respectively, malignant hyperthermia and hyperkalaemic periodic paralysis in homozygotes, yet increased muscle mass in heterozygotes [12],[13], or of a *FGFR3* mutation causing hereditary chondrodysplasia in homozygous sheep and increased size in the carriers [14],[15].

A diagnostic test for the CTS mutation has been developed and already applied on more than 4,000 BBCB samples. The resulting information should have an immediate and positive impact on the incidence of CTS, and protect animals and breeders against the pathological condition and ensuing economic losses.

This work is yet another illustration of the value of domestic animal populations in enriching the phenotype-genotype map. It adds to a recent list of positional cloning successes in poultry [16], dog [17],[18], horse [19] and bovine [2].

## Materials and Methods

### Mutation scanning

Coding exons of positional candidate genes were amplified from genomic DNA of a CTS case and a matched control using standard procedures. The primers used for the *MRC2* gene are listed in Table S3. PCR products were directly sequenced using the Big Dye terminator cycle sequencing kit (Applied Biosystem, Foster City, CA). Electrophoresis of purified sequencing reactions was performed on an ABI PRISM 3730 DNA analyzer (PE Applied Biosystems, Foster City, CA). Multiple sequence traces from affected and wild-type animals were aligned and compared using the Phred/Phrap/Consed package (www.genome.washington.edu).

### 5′ exonuclease diagnostic assay of the CTS mutation

A Taqman assay was developed to genotype the CTS mutation, using 5′-GCG CAA CAG CAC CAG AGA-3′ and 5′-CTC CCT ACC TTG TTC AGG AAC TG-3′ as PCR primers, and 5′-CTG CCG CCC AC[* *] GGG-3′ (CTS) and 5′-CTG CCG CCC AC[**A G**]G-3′ (wild type) as Taqman probes. Reactions were carried out on a ABI7900HT instrument (Applied Biosystems, Foster City, CA) using standard procedures.

### Allelic imbalance test of NMD

Total RNA was extracted from lung, heart and skeletal muscle of a two month old heterozygote *c.2904_2905delAG* animal using Trizol (Invitrogen). The RNA was treated with TurboDNase (Ambion). cDNA was synthesized using *SuperscriptTMIII* First Strand Synthesis System for RT-PCR (Invitrogen). A portion of *MRC2* cDNA, encompassing the deletion, was amplified using *MRC2* specific primers (Table S4). The PCR products were directly sequenced as described above.

### Real-time quantitative RT-PCR test of NMD

Total RNA from lung and skeletal muscle was obtained from animals of the three genotypes (*+/+*, *+/CTS* and *CTS/CTS*). After *DNase*-treatment (Turbo DNA-free, Ambion), 500 ng total RNA was reverse transcribed in a final volume of 20 µl using the iScript cDNA Synthesis Kit (Bio-Rad). PCR reactions were performed in a final volume of 15 µl containing 2 µl of 2.5-fold diluted cDNA (corresponding to 20 ng of starting total RNA), 7.5 µl of 2× master mix prepared from the qPCR Core Kit for SYBR green I (Eurogentec), 0.45 µl of 1/2000 SYBR green I working solution prepared from the qPCR Core Kit for SYBR green I (Eurogentec), forward and reverse primers (250 nM each) and nuclease free water. PCRs were performed on a an ABI7900HT instrument (Applied Biosystems, Foster City, CA) under the following cycling conditions: 10 min at 95°C followed by 40 cycles at 95°C for 15 sec and 60°C for 1 min. Two primer sets were used to test *MRC2* expression (MRC2_5′QRT_UP/DN and MRC2_3′QRT_UP/DN) and seven genes were included as candidate endogenous controls: (1) Beta Actin *(ACTB)*, (2) Glyceraldehyde-3-phosphate Dehydrogenase (*GAPD*), (3) Hypoxanthine Phosphoribosyltransferase 1 *(HPRT1)*, (4) Ribosomal Protein Large P0 *(RPLP0)*, (5) Ribosomal Protein S18 *(RPS18)*, (6) Succinate Dehydrogenase Complex Subunit A Flavoprotein *(SDHA)*, and (7) Tyr-3- & Trp-5-Monooxygenase Activation Protein Beta *(YWHAB)*. After analyse of the results with geNorm [20], the four following genes were selected as best endogenous controls: *ACTB*, *RPLP0*, *RPS18* and *YWHAB*. The corresponding primer sequences are given in Table S4. All sample/gene combinations were analyzed in triplicate. Relative *MRC2* expression levels, for the 5′ & 3′cDNA parts, in the samples of the three genotypes were computed using the qBase software package (http://medgen.ugent.be/qbase/)(Hellemans et al., 2007).

### Western blotting

A series of available antibodies directed against the human Endo180 were tested by Western blotting for cross reactivity with bovine Endo180 on commercial bovine aortic endothelial cells (BAOEC, Cell Applications). A positive control corresponding to a lysate of MRC5 human fibroblast cell line expressing Endo180 was included in each experiment. The tested antibodies were the following: (i) seven mouse monoclonal antibodies (for details see [21]–[23]), (ii) a rabbit polyclonal antibody (DEX) directed against the full length human protein [21] and (iii) two rabbit polyclonal antibodies (CAT1 and CAT2) against a peptide from the human C-terminal cytoplasmic domain (CATEKNILVSDMEMNEQQE) conjugated to KLH [6]. After initial testing, only the CAT2 antibody was retained for further experiments. Flash-frozen skeletal muscle and lung tissues from animals of the three *MRC2* genotypes (see above) were disrupted and homogenized with a tissue lyser system II (Quiagen). Crude protein extracts were obtained and total protein concentrations determined using a colorimetric test (Pierce BCA Protein Assay kit, Thermo Scientific). Fifteen µg were diluted in 15 µl final volume (1× SDS gel-loading buffer) and loaded on a 5% stacking – 10% resolving Tris-glycine SDS-Polyacrylamide gel. Proteins were separated by electrophoresis at 120 V-250 mA during 3 hours, visualized by Coomassie blue staining, and electro-transferred overnight to Hybond P PVDF membranes (GE Healthcare). Membranes were blocked with 5% skim milk in PBS-Tween 20 (PBS-T) followed by incubation with primary CAT2 antibodies (1∶200) in a total volume of 3 ml for 1 h30 min. After washing, the specific signal was detected by using Alkaline Phosphatase conjugated secondary rabbit antibodies (Sigma) following the instructions of the manufacturer.

### Statistical analysis

Phenotypes corresponded to 22 type traits related to muscularity, skeletal conformation, size and leg soundness that are systematically recorded in the BBCB [24]. These were analyzed using a mixed model including genotype at the *MRC2* locus (2), year at scoring (2), body condition (4) as fixed effects, age at scoring as covariate (quadratic regression), the additive genetic animal effect and the residual effect as random effect [25]. The number of animals in the relationship matrix was 6,356. Variance components were estimated using the DFREML method (Derivative-Free Restricted Maximum Likelihood) [26]. The part of the genetic variance due to *MRC2* genotype was estimated as the difference between the variance due to the animal model with and without *MRC2* genotype in the model. The allele substitution effects (contrast) were calculated as the difference between the genotypic means (+/+ and +/M) obtained from the mixed model equations.

### Evidencing a selective sweep

We simulated the segregation of a heterozygous mutation from Précieux to its 160 [2003–2005] sire offspring. Variable parameter values were (i) the transmission rate of the mutation from carriers to their offspring (0.5 to 0.75), (ii) the frequency of the mutation outside of the Précieux lineage. 10,000 simulations were conducted for each set of parameter values. Only non-affected genotypes were sampled from matings between heterozygous parents.

## Supporting Information

Table S1 Statistics of number of carriers under the neutral model (no selection)(10,000 simulations).
(0.07 MB PDF)

Table S2 The table shows, for varying values of δ, the proportion of the phenotypic (P-PV) and genetic variance (P-GV) explained by the QTN in the general population. Assume a normally disturbed trait with 25% heritability, influenced by a QTN with MAF 1 of 0.25 and with two possible genotypes in the population (+/+ and +/M) as is the case for the CTS mutation. Assume that the average phenotype of the +/+ population is -δ/2 and of the +/M population is +δ/2. Assume also that the residual variance is 1. The table shows, for varying values of δ, the proportion of the phenotypic (P-PV) and genetic variance (P-GV) explained by the QTN in the general population. Assume that one selects future AI sires amongst offspring of popular +/M heterozygous sires. The table shows, for five hypothetical phenotypic threshold values for selection T = 1.00–2.00, the proportion of sons selected (Prop-Sel), and amongst the selected sons, the ratio of carrier (+/M) versus non-carriers (+/+) (C/NC). Dams were assumed to be +/+ for simplicity. It can be seen that the observed ∼2∶1 segregation ratio observed for CTS implies a selection intensity of the order of 0.02 for a QTN that accounts for ∼0.05 of the genetic variance in the general population. The corresponding cells are highlighted in gray.
(0.11 MB PDF)

Table S3 Primer pairs for the *MRC2* gene.
(0.08 MB PDF)

Table S4 Allelic imbalance and quantitative RTPCR primer pairs for the detection of NMD.
(0.08 MB PDF)
