# Supplementary material for: Balancing Selection of a Frame-Shift Mutation in the MRC2 Gene Accounts for the Outbreak of the Crooked Tail Syndrome in Belgian Blue Cattle
Source: PLoS Genet. 2009 Sep 25;5(9):e1000666. doi: 10.1371/journal.pgen.1000666 (PMC2739430; doi:10.1371/journal.pgen.1000666)
Supplement: Table S1 — Statistics of number of carriers under the neutral model (no selection)(10,000 simulations). (0.07 MB PDF) [file pgen.1000666.s001.pdf]

Supplemental Table 1: Statistics of number of carriers under the neutral model (no selection)(10,000 simulations).

f_{mut}	Min	25%	median	mean	75%	max	n>44
0.00	0	2	7	9.54	15	62	14
0.01	0	4	10	11.76	17	58	23
0.05	0	13	19	20.12	26	63	130
0.10	4	22	29	29.41	36	72	688
