# Supplementary material for: Balancing Selection of a Frame-Shift Mutation in the MRC2 Gene Accounts for the Outbreak of the Crooked Tail Syndrome in Belgian Blue Cattle
Source: PLoS Genet. 2009 Sep 25;5(9):e1000666. doi: 10.1371/journal.pgen.1000666 (PMC2739430; doi:10.1371/journal.pgen.1000666)
Supplement: Table S2 — The table shows, for varying values of δ, the proportion of the phenotypic (P-PV) and genetic variance (P-GV) explained by the QTN in the general population. Assume a normally disturbed trait with 25% heritability, influenced by a QTN with MAF 1 of 0.25 and with two possible genotypes in the population (+/+ and +/M) as is the case for the CTS mutation. Assume that the average phenotype of the +/+ population is -δ/2 and of the +/M population is +δ/2. Assume also that the residual variance is 1. The table shows, for varying values of δ, the proportion of the phenotypic (P-PV) and genetic variance (P-GV) explained by the QTN in the general population. Assume that one selects future AI sires amongst offspring of popular +/M heterozygous sires. The table shows, for five hypothetical phenotypic threshold values for selection T = 1.00–2.00, the proportion of sons selected (Prop-Sel), and amongst the selected sons, the ratio of carrier (+/M) versus non-carriers (+/+) (C/NC). Dams were assumed to be +/+ for simplicity. It can be seen that the observed ∼2∶1 segregation ratio observed for CTS implies a selection intensity of the order of 0.02 for a QTN that accounts for ∼0.05 of the genetic variance in the general population. The corresponding cells are highlighted in gray. (0.11 MB PDF) [file pgen.1000666.s002.pdf]

Supplemental table 2: Assume a normally disturbed trait with 25% heritability, influenced by a QTN with MAF of 0.25 and with two possible genotypes in the population (+/+ and +/M) as is the case for the CTS mutation. Assume that the average phenotype of the +/+ population is -δ/2 and of the +/M population is +δ/2. Assume also that the residual variance is 1. The following table shows, for varying values of δ, the proportion of the phenotypic (P-PV) and genetic variance (P-GV) explained by the QTN in the general population. Assume that one selects future AI sires amongst offspring of popular +/M heterozygous sires. The following table shows, for five hypothetical phenotypic threshold values for selection (T=1,00-2,00), the proportion of sons selected (Prop-Sel), and amongst the selected sons, the ratio of carrier (+/M) versus non-carriers (+/+) (C/NC). Dams were assumed to be +/+ for simplicity. It can be seen that the observed ~2:1 segregation ratio observed for CTS implies a selection intensity of the order of 0,02 for a QTN that accounts for ~0,05 of the genetic variance in the general population. The corresponding cells are highlighted in gray.

QTN	Population		1/2-sib pedigrees - carrier sire									
			T = 1,00		T = 1,25		T = 1,50		T = 1,75		T = 2,00	
δ	Prop-PV	Prop-GV	Prop-Sel	C/NC	Prop-Sel	C/NC	Prop-Sel	C/NC	Prop-Sel	C/NC	Prop-Sel	C/NC
0,00	0,00	0,00	0,15	1,00	0,10	1,00	0,06	1,00	0,04	1,00	0,02	1,00
0,05	0,00	0,00	0,15	1,08	0,10	1,10	0,06	1,11	0,04	1,12	0,02	1,13
0,10	0,00	0,01	0,15	1,17	0,10	1,20	0,06	1,23	0,04	1,26	0,02	1,29
0,15	0,00	0,02	0,15	1,27	0,10	1,31	0,06	1,36	0,04	1,41	0,02	1,46
0,20	0,01	0,03	0,15	1,38	0,10	1,44	0,06	1,51	0,04	1,58	0,02	1,65
0,25	0,01	0,05	0,15	1,49	0,10	1,58	0,06	1,67	0,04	1,77	0,02	1,87
0,30	0,02	0,07	0,15	1,62	0,10	1,73	0,06	1,85	0,04	1,98	0,02	2,12
0,35	0,02	0,09	0,15	1,75	0,10	1,89	0,06	2,05	0,04	2,22	0,02	2,41
0,40	0,03	0,12	0,16	1,90	0,10	2,07	0,06	2,27	0,04	2,49	0,02	2,73
0,45	0,04	0,15	0,16	2,06	0,10	2,27	0,07	2,51	0,04	2,79	0,02	3,10
0,50	0,04	0,18	0,16	2,23	0,11	2,49	0,07	2,78	0,04	3,12	0,02	3,51
0,55	0,05	0,21	0,16	2,42	0,11	2,72	0,07	3,08	0,04	3,50	0,02	3,98
0,60	0,06	0,25	0,16	2,62	0,11	2,98	0,07	3,41	0,04	3,92	0,02	4,51
0,65	0,07	0,29	0,16	2,84	0,11	3,27	0,07	3,78	0,04	4,39	0,02	5,12
0,70	0,08	0,34	0,17	3,08	0,11	3,58	0,07	4,19	0,04	4,92	0,03	5,81
0,75	0,10	0,38	0,17	3,33	0,11	3,92	0,07	4,64	0,05	5,52	0,03	6,58
0,80	0,11	0,43	0,17	3,61	0,12	4,30	0,08	5,15	0,05	6,19	0,03	7,47
0,85	0,12	0,48	0,17	3,92	0,12	4,71	0,08	5,70	0,05	6,93	0,03	8,47
0,90	0,13	0,53	0,18	4,25	0,12	5,16	0,08	6,32	0,05	7,77	0,03	9,60
0,95	0,14	0,58	0,18	4,60	0,12	5,66	0,08	7,00	0,05	8,71	0,03	10,89
1,00	0,16	0,63	0,18	4,99	0,13	6,20	0,09	7,76	0,05	9,77	0,03	12,35
