# Supplementary material for: Balancing Selection of a Frame-Shift Mutation in the MRC2 Gene Accounts for the Outbreak of the Crooked Tail Syndrome in Belgian Blue Cattle
Source: PLoS Genet. 2009 Sep 25;5(9):e1000666. doi: 10.1371/journal.pgen.1000666 (PMC2739430; doi:10.1371/journal.pgen.1000666)
Supplement: Table S3 — Primer pairs for the MRC2 gene. (0.08 MB PDF) [file pgen.1000666.s003.pdf]

Supplemental table 3: Primer pairs for the *MRC2* gene

Name	Primer sequence (5'-3')	Gene part	Size (bp)
gUP1	CCGGAGGAAGACGCGAGCCCCT	exon 1 (ATG)	327
gDN1	GGGGGAAAGGAGGAAAAAGTCG		
gUP2	CACAGCCCACTACCAGCGTCAG	exon 2	610
gDN2	CCATGACGATGAAAGAGCTGAC		
gUP3	ACCCTGTGAGAAGCCTTTCCTG	exons 3, 4	907
gDN3	GATGTAGGTCTGCTCGTGGATC		
gUP4	TGTCATGGTGGCAGGTAACGAC	exons 4, 5	576
gDN4	GGGTGGAAATCTGCTGGTCTAG		
gUP5	GGAGGAGGCAAGAGAGCCGAAG	exon 6	366
gDN5	CCTTGTGCTGTGAGGGTGGGTG		
gUP6	AAAGCGTGGTCCCTGTCCCAGC	exon 7	813
gDN6	AACGGTAGCACTCCTTTGGTGGT		
gUP7	GGCTTGGTGGGAAGAGTGGATCT	exons 8, 9	725
gDN7	GGGGAGGAGGGATTCCGAGAGG		
gUP8	TTGGAGGCATCTGCACAGCTAC	exon 10	407
gDN8	TACCACAGGAGGCTGCGGATTC		
gUP9	CCTGTGCTCAAGCCTGCAGAAA	exon 11	375
gDN9	GCCCTGGAGATAGTTGAAGCTCA		
gUP10	GGTCCCCACTTCCCTGAGCAAG	exon 12	375
gDN10	ATGAAGCCCTAGGTCTCGGTCAT		
gUP11	CAACCCCACAGCACATGTCCCT	exon 13	449
gDN11	CTGCTCGGATCATGGCTGGGTC		
gUP12	CCTCGACACCCTGTCCACTGAA	exon 14	398
gDN12	CCTGGCACTAGCAGCAGACACA		
gUP13	CCCGAGTCACAACTGCAAGGATT	exons 15, 16, 17	751
gDN13	GGGTGTGGGATGGACAGGAAGC		
gUP14	CTGCAGCGTGTCTGTCCCTGTT	exon 17, 18	697
gDN14	CGAGTCCCTGCTAGCCATCCAC		
gUP15	GGTCTAACCTGGTGCCCTGTACT	exons 19, 20 (*)	693
gDN15	AGGGGAGAGGGTGGTAGGTTCAG		
gUP16	GCCAGGTCGGGAGGGTATCAGAG	exons 21, 22, 23	1042
gDN16	CTGCAACCCCTGGATGCTCACT		
gUP17	GAGTTGGTCTCTGCCTGCTGTTC	exons 24, 25, 26	995
gDN17	CAGAGTGCAGCACGGGGACTATA		
gUP18	CGTCTCCATGCCATCCTCTATTC	exons 27, 28	743
gDN18	CCCAGGCCTCCCATCCACTGTG		
gUP19	ACTGTATTGTTACTACCACTGTTGTT	exons 29, 30 (STOP)	610
gDN19	AAGGAAACGCCATGCTGCACTC		
