# Supplementary material for: Balancing Selection of a Frame-Shift Mutation in the MRC2 Gene Accounts for the Outbreak of the Crooked Tail Syndrome in Belgian Blue Cattle
Source: PLoS Genet. 2009 Sep 25;5(9):e1000666. doi: 10.1371/journal.pgen.1000666 (PMC2739430; doi:10.1371/journal.pgen.1000666)
Supplement: Table S4 — Allelic imbalance and quantitative RTPCR primer pairs for the detection of NMD. (0.08 MB PDF) [file pgen.1000666.s004.pdf]

Supplemental table 4: Allelic imbalance and quantitative RT-PCR primer pairs for the detection of NMD.

Gene	Forward primer	Reverse primer	Size (bp)
MRC2 del	GACAAGAAGTGCGTGTACATGATG	AGAACTGTGCCTCTGACCACTTC	232
MRC2 5’ part	CGAGTCTCTCCAGCCTGCAATG	ACTCAGTGCCTCGCGGTCACAC	168
MRC2 3’ part	TCCTGCTCCTGGCTCTGCTGAC	CTGCTGCTCGTTCATTTCCATG	167
ACTB	TCGCGGACAGGATGCAGAAAGA	GCTGATCCACATCTGCTGGAA	149
GAPDH	TGACCCCTTCATTGACCTTCA	GATGGTGATGGCCTTTCCATT	127
HPRT1	TGCTGAGGATTTGGAGAAGG	CAACAGGTCGGCAAAGAACT	154
RPLP0	TGGGCAAGAACACGATGATG	TGAGGTCCTCCTTGGTGAACA	123
RPS18	GCAGAATCCACGCCAATACAA	TCTTCAGGCGCTCCAGGTCTTC	135
SDHA	GCAGAACCTGATGCTTTGTG	CGTAGGAGAGCGTGTGCTT	185
YWHAZ	GCATCCCACAGACTATTTCC	GCAAAGACAATGACAGACCA	120
